# Evaluation of Person-Centredness in Rehabilitation for People Living with Dementia Is Needed: A Review of the Literature

**DOI:** 10.1155/2019/8510792

**Published:** 2019-05-02

**Authors:** Kate Allen Christensen, Karen-Margrethe Lund, Jette Thuesen

**Affiliations:** ^1^Department of Occupational Therapy and Physiotherapy, Zealand University Hospital, Roskilde–Koege, Sygehusvej 10, 4000 Roskilde, Denmark; ^2^Department of Public Health, J. B. Winsløwsvej 9A, University of Southern Denmark, 5000 Odense C, Denmark; ^3^Department of Occupational Therapy, University College Absalon, Parkvej 190, 4700 Næstved, Denmark; ^4^Danish Knowledge Centre for Rehabilitation and Palliative Care (REHPA), Vestergade 17, 5800 Nyborg, Denmark

## Abstract

**Background:**

With an expected increase in the prevalence of dementia, change in care policies and healthcare systems worldwide is needed. Rehabilitation is increasingly recognised as contributing to dementia care. Rehabilitation subscribes to person-centredness, and thus, evaluations of person-centredness in rehabilitation for people living with dementia are relevant in order for healthcare professionals to know how best to practice person-centredness.

**Aim:**

The aim of this study was to identify methods of evaluating person-centeredness in rehabilitation for people living with dementia.

**Materials and Methods:**

Review of the literature using the search terms *dementia*, *person-centredness*, and *rehabilitation or occupational therapy*. Databases searched included: CINAHL, PubMed, Embase, PsycINFO, OTseeker, and SveMed+. The study included peer-reviewed articles from year 2000 to 2018 in Danish, English, Norwegian, or Swedish.

**Results:**

Only one academic article met the inclusion criteria. In that article, person-centred practice was evaluated using observation and interview as well as analytical frameworks from person-centred care and occupational therapy.

**Conclusion:**

Evaluations of person-centred practice in rehabilitation for people living with dementia in peer-reviewed literature are lacking. Evaluations are needed to identify effective strategies to pursue and uphold person-centred care. Given the dearth of research on evaluations of person-centredness in rehabilitation, this article included research in person-centred dementia care in the discussion, which potentially can inspire practice and research of rehabilitation for people living with dementia. To understand the complex nature of person-centredness, a variety of research methodologies of qualitative and quantitative characters are recommended for evaluations.

## 1. Introduction

According to the World Health Organisation (WHO), 35.6 million people across the world live with a dementia disease [1]. Dementia is a clinical syndrome characterised by progressive cognitive decline that interferes with the ability to perform daily activities and live independently [1, 2]. The increase in the number of people living with dementia necessitates change in care policies and healthcare systems to maintain acceptable standards of care and quality of life for people living with dementia [3]. Rehabilitation is increasingly recognised as contributing to dementia care [4–7]. Rehabilitation appears as a core recommendation in the recent World Health Organisation Global Action Plan on the Public Health Response to Dementia [8], and in Denmark, rehabilitation is recommended in national clinical guidelines [9].

According to Linda Clare [5], a leading scholar in the field, the rehabilitation philosophy is genuinely person-centred and reflects important values underpinning good dementia care. Clare associates person-centredness in dementia rehabilitation to the theoretical works of Tom Kitwood [10]. In dementia care, person-centredness includes supporting individual personhood and establishing meaningful relationships, shared decision making, and personalised care and environments, using the person's biography [10, 11].

Person-centred rehabilitation for people living with dementia involves working with people to achieve the goals that are important to them, acknowledging that each individual has a unique set of experiences, values, motivations, strengths, and needs [5]. Also, in generic rehabilitation literature, person-centredness has been described as a key principle and value [12, 13].

Conceptually, person-centredness has much in common with the concept of *client-centredness* underpinning occupational therapy [14, 15]. Person-centredness also overlaps with *patient engagement* in healthcare, comprising similar features such as shared decision making and individually tailored interventions [16]. In this study, we use person-centredness as an umbrella concept. Following Hughes and colleagues [17] we consider the idea of *centredness* to comprise issues related to respect for individuality, personal values and meaning, therapeutic alliance, social context and relationships, inclusive model of health and wellbeing, expert lay knowledge, shared responsibility, communication, autonomy, and the professional as a person.

Research on person-centredness is growing, showing challenges and potentials in rehabilitation [18, 19], occupational therapy [20], dementia care [21, 22], and healthcare in general [16, 23]. Barriers to a person-centred approach in healthcare for people with dementia include some healthcare professionals doubting the capacity of people living with dementia to partake in decision making [24, 25]. Conversely, other researchers argue that people living with dementia want to be involved in making decisions about their own care, e.g., through individualised care plans [26, 27].

As healthcare worldwide is advocating evidence-based practice to assure a sound knowledge base for interventions, it is relevant to investigate and evaluate person-centred approaches. A review of the literature on people with dementia and family involvement in shared decision making showed that people with dementia were involved in decision making to various degrees, but most were prematurely excluded from decision making [25]. A later meta-analysis concluded that intensive person-centred care for people with dementia improved their neuropsychiatric symptoms and quality of life in long-term care but that future research should include how person-centred care is carried out in daily practice [28].

A recent review on person-centred care for individuals with dementia argued the need for evaluating care practices to make appropriate changes to person-centred care [29]. Person-centeredness in rehabilitation might encompass other elements than person-centeredness in other care contexts [30]. Considering the focus of person-centredness in rehabilitation as well as occupational therapy, we were curious to explore how person-centredness was evaluated in these fields of practices.

The aim of this study was to identify methods of evaluating person-centeredness in rehabilitation for people living with dementia.

## 2. Materials and Methods

We used the principles presented by Gough et al. [31] to understand and guide reflections of the review of the literature. The matrix method [32], as well as the 27-item checklist of PRISMA Statement Explanation and Elaboration document [33], was used as structured guides to organise and conduct the review.

## 3. Search Strategy

To generate the list of search terms, we undertook preliminary searches in relevant scholarly databases to identify subject headings and keywords, as recommended by Lund et al. [34]. Articles from the preliminary searches, as well as experts in the field of dementia and rehabilitation, further qualified the list of search terms. On the 14th of March 2017, the following databases were searched using subject headings (e.g., CINAHL headings and MeSH) and keywords on the search terms *dementia, person-centredness, and rehabilitation or occupational therapy*: CINAHL via EBSCOhost, PubMed via NCBI, Embase and PsycINFO via Ovid, OTseeker, and SveMed+ (CINAHL and PubMed search strategies are detailed in Appendix 1). Limitations included peer-reviewed articles published between 2000 and 2017 and in English, Swedish, Norwegian, or Danish. Email alerts from the databases were received until 1st of November 2018. OTseeker and SveMed+ were re-searched on the 19th of November 2018, as these databases do not provide alert services.

### 3.1. Inclusion Criteria

- Peer-reviewed articles in English, Danish, Norwegian, or Swedish published from 2000 to November 2018, as prominent authors in the fields of rehabilitation of people living with dementia initiated research around 2000 [35].
- Participants of all ages, with a diagnosis of dementia, including Alzheimer's, Lewy body disease, vascular dementia, or frontotemporal dementia, as the most common underlying pathologies of dementia [1, 2].
- Studies evaluating aspects of person-centredness: respect for individuality, personal values and meaning, therapeutic alliance, social context and relationships, inclusive model of health and wellbeing, expert lay knowledge, shared responsibility, communication, autonomy, and the professional as a person [17].
- Studies using all forms of methodological design to evaluate person-centredness. We defined evaluation as any method (e.g., interview, observation, and questionnaire) that collected and documented information on person-centredness [18].
- Settings of rehabilitation or occupational therapy, as occupational therapy is often part of rehabilitation and a recommended discipline for people living with dementia [36–38], and client-centred practice forms the basis of occupational therapy [15].

### 3.2. Exclusion Criteria

- Study participants with a diagnosis of mild cognitive impairment, Huntington's disease, Creutzfeldt Jacobs, dementia associated with Parkinson's, and AIDS or cognitive decline not diagnosed as dementia
- Interventions focused on person-centredness in research or participatory design
- Approaches aimed exclusively at the next of kin or informal caregiver
- Study protocols and literature reviews
- Studies from nonwestern countries, to utilise knowledge from countries with which Denmark usually compares itself

## 4. Study Selection

Two authors searched and reviewed the literature (KML and KAC). Both carried out the preliminary searches, identifying subject headings and keywords. One reviewer (KAC) did the final search and exported the results to the EndNote reference system [39]. Duplicates were checked electronically and manually in Endnote by both reviewers. Articles were exported from Endnote to the screening and data extraction tool Covidence [40]. Each reviewer independently screened titles and abstracts in Covidence. The inclusion and exclusion criteria guided the title and abstract screening.

Inspired by Garrard [32], a review matrix was created in an Excel spreadsheet that contained our research questions. Both reviewers read the articles included for full-text reading and each reviewer filled out the review matrix independently and together discussed the inclusion, until consensus was reached [33]. A checklist from the Critical Appraisal Skills Programme (CASP) was used to assess the quality of final inclusion [41].

## 5. Results

**Figure 1:**
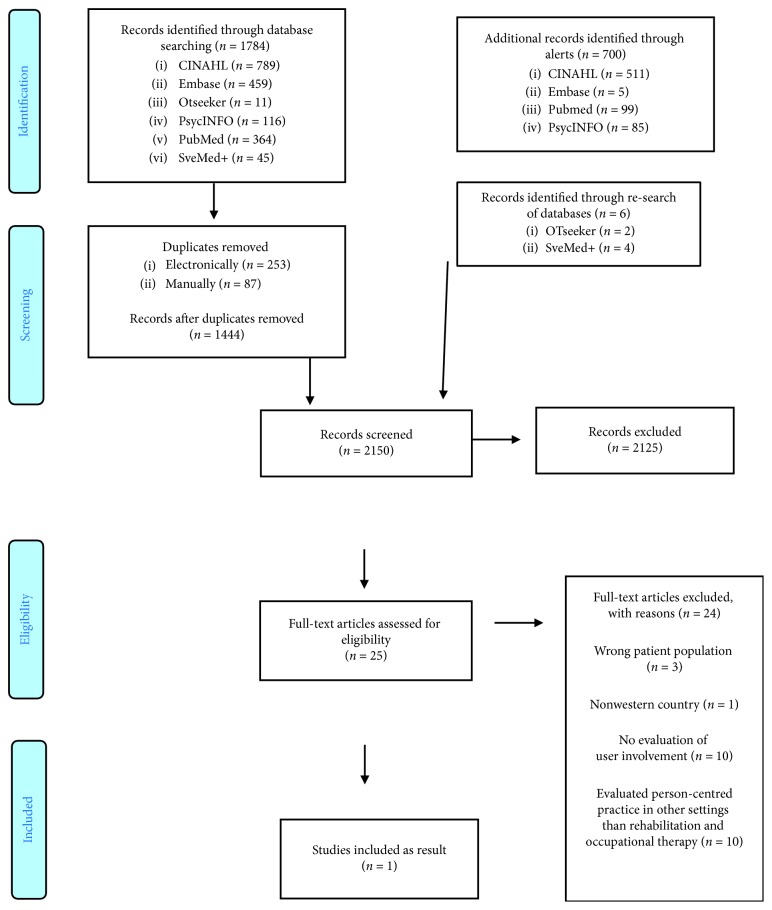
PRISMA 2009 flow diagram.

The result of the search strategy is presented in a PRISMA flow diagram in Figure 1 [42]. A total of 2150 articles were identified, 1444 articles via databases and 706 articles via database alerts and re-searching. Titles and abstracts were screened by both reviewers on 2150 peer-reviewed articles. Full texts were read for further assessment of the eligibility of 25 articles, of which only one article met the criteria for inclusion. Articles were mostly excluded, as evaluation of person-centredness was lacking or person-centredness was evaluated in other settings than rehabilitation and occupational therapy.

### 5.1. Quality of the Included Article

The quality of the study was assessed using a checklist for qualitative studies, from CASP [43]. As the result study referred to Raber et al. [44] for methodological details, information from this study was included. There was a clear statement of the aim of the research, including goals, importance, and relevance. The aim was to present and discuss the potency of the social environment in promoting volition and engagement in people living with dementia, using two case studies, each including two people with moderate dementia, two family members, and two healthcare professionals. The setting was therapeutic activity sessions in occupational therapy in a memory support unit [45]. Qualitative methods of observations of people living with dementia and healthcare professionals were used, as well as interviews with healthcare professionals and families [45]. Person-centredness was evaluated using analytical frameworks derived from person-centred care and occupational therapy [46, 47].

The methodological design of phenomenology was appropriate to address the aim of examining participants lived experience. Inclusion criteria were presented; however, it was not clear if some people chose not to take part in the study. Generally, transparency in data collection was present, with a topic guide for participant observation; however, a topic guide for the interviews lacked. Analysis showed rigour in generation of themes across cases. Ethics were considered, and consent forms were completed for all participants; however, there were no details on how research was explained to participants. There was a clear statement of findings and discussion of the evidence and credibility of the findings (Table 1).

### 5.2. Characteristics of the Included Article

Person-centred practice was evaluated using observation and interview as well as analytical frameworks from person-centred care and occupational therapy. The result of the included article was that social therapeutic interactions can promote or inhibit occupational engagement. The social environment was analysed in terms of the role of staff in providing an environment that promoted volitional expressions and occupational engagement. The focus was on healthcare professionals' abilities to interact and communicate with people with dementia. The study showed that people living with dementia maintained a desire to engage in everyday activities, but if healthcare professionals were unskilled in identifying or overruled clients' efforts, the level of engagement was lowered. Communication skills for promoting volitional expressions and occupational engagement included aspects of recognition, validation, negotiating personal preferences, celebrating activities of enjoyment, capitalising on remaining interests and strengths, and encouraging and reinforcing engagement. Attention was given to fluctuating abilities and elusive ways of expressing preferences. The preferences of people living with dementia were typically indicated, not through verbal or behavioural movement towards activities but rather through behaviours demonstrating resistance to participate. Therefore, fine-tuned observational skills and the use of observational assessments were important as well as abilities to reflect on the therapeutic use of self [45].

To provide a more person-centred social environment the authors highlighted (1) a substantial need for education with a focus on skills of communication and observation, including reflection for both staff and family and (2) culture change in the facility [45].

## 6. Discussion

The aim of this study was to identify methods of evaluating person-centeredness in rehabilitation for people living with dementia. Considering the focus on person-centredness and rehabilitation for people living with dementia, it was surprising to find that only one study was found in a review of the literature that evaluated person-centredness in rehabilitation for people living with dementia. Moreover, the article did not explicitly address rehabilitation, but occupational therapy.

Person-centredness includes among other components *social context and relationships* [17]. The included study focused on the social environment in terms of interaction, communication, and observation. Following these findings, we will discuss the social environment, communication and interaction, and the use of observational skills in understanding people living with dementia. We will suggest potential ways to evaluate person-centred practice in rehabilitation based on literature from other fields of practices. Finally, we will reflect on the concept of rehabilitation for people living with dementia.

### 6.1. Social Environment

Teitelman, Raber, and Watts [45] focused on the significance of the social environment in determining whether people with dementia engage in preferred occupations. A recent critical interpretive synthesis of meaningful engagement and person-centred residential dementia care concluded similar findings, highlighting that collaborative partnerships between staff, residents, family members, and significant others were critical in implementing person-centred care [48]. According to empirical studies from other fields of practice, not only the social but also the organisational environment like policies, leadership, routines, architecture, and shared accommodation can promote or restrict person-centred approaches [49–51]. Inadequate staffing can result in task-oriented care instead of person-centred care, and participation of people living with dementia can be adjusted, primarily to suit institutional objectives and secondly to fit the resident's needs and wishes [52, 53]. St-Amant et al. [51] revealed how the Canadian homecare system based decisions related to moving into nursing homes on waiting lists and not on the expressed needs of people living with dementia. According to other studies, involvement of residents with dementia can be enhanced if leaders are role models and provide healthcare professionals with support, acknowledgement, and feedback on their interactional abilities [49, 50]. Similar challenges and potentials are raised in OT literature [54, 55] and in literature on healthcare in general [56].

### 6.2. Communication and Interaction

The potential power dilemmas in communication and interaction described by Teitelman et al. [45] are supported in research from other fields of practice and discussed as a barrier to person-centredness [57, 58]. Studies describe care climates, where healthcare professionals dehumanise people living with dementia as people who do not know their own best interest [53, 57].

Similar to Teitelman et al. [45], other researches describe a variety of ways to interact and communicate with people living with dementia [57, 59]. Being fully present and using skills such as empathy, advocacy, and patience may influence the ability and wishes of people living with dementia to participate [50, 60]. The need for healthcare professionals to understand a person's motivation to engage in activities is especially important as illness progresses, where facilitation of engagement in alternative everyday activities may be necessary [61]. It is here relevant to consider that individual preferences might change over time and there is therefore a risk that inaccurate assumptions about the preferences of people living with dementia can be made, if not first reflecting or clarifying with the person themselves [48, 52].

Teitelman et al. [45] noted that communication was challenged by fluctuating abilities of people living with dementia to express their preferences and take part in shared decision making. This has also been observed in other studies [51, 52]. Using models like *the Intentional Relationship Model* may make professionals aware of the therapeutic use of self and foster interpersonal encounters [45, 47].

### 6.3. Use of Observation in Understanding People with Dementia

Teitelman et al. [45] described the need for observational skills in communication and in understanding the preferences of people living with dementia. They found that the preferences of people living with dementia were often expressed through behaviours demonstrating a desire not to participate. This embodied way of communication is challenging for healthcare professionals to routinely identify [62]. Drawing on the idea of *embodied personhood*, Kontos and Naglie [62] advocated the communicative capacity of the body to connect people to each other, fostering sympathetic care and improving person-centred dementia care. Especially when people with dementia show signs of severe cognitive impairment, healthcare professionals may better achieve person-centred care when recognising that personhood persists despite the presence of cognitive impairment. This is, for example, achieved when professionals observe and imagine how another person might feel in a given situation, based on their own bodily experiences [62].

To further understand people living with dementia, it is relevant to observe the person's engagement in everyday activities [63, 64]. A literature review found that people living with dementia want to engage in meaningful activities to be connected with self, others, and the environment [61]. In a study of meanings and motives for engagement in self-chosen occupation, it was found that selecting occupations might contribute to personal identity, experience of autonomy, and increased wellbeing of people living with dementia [63]. Research has shown how everyday activities, like meal times, self-care, or music activities, can be altered to become therapeutic interactions with high levels of engagement and decision making [52, 59].

### 6.4. Need for Evaluation

As only one study was found in this review of the literature that evaluated person-centredness in rehabilitation, we argue that there is a need for further research that evaluates person-centred approaches in rehabilitation for people living with dementia. This need is also pertinent in person-centred dementia care in general [29]. Although rehabilitation and occupational therapy subscribe to person-centredness, it is not imperative that all healthcare professionals working in rehabilitation or as occupational therapists practice person-centredness [54, 65]. Evaluations of person-centred practice are important, in order for person-centredness to continue to be a guiding principle in rehabilitation and occupational therapy [20].

Future research in person-centred rehabilitation can learn from research in person-centred dementia care in general. In their study, Teitelman and colleagues [45] used observational methods as well as interviews with healthcare professionals and families. A combination of interview and observational methods are widely used when evaluating person-centred care in dementia [50, 52], including the observation of interactions in daily activities [50, 52]. Observations can be guided by existing standardised assessment procedures like Dementia Care Mapping [60] or by qualitative methodology such as grounded theory [50, 53] or ethnography [51, 57]. Edvardsson, Sandman, and Borell [49] used the Swedish version of person-centred care assessment tool (PCAT) and the person-centred climate questionnaire (PCQ) to measure perceived person-centredness of care and environment from the perspectives of healthcare professionals. Perceived person-centredness of care could also be evaluated by residents and family members [49].

Teitelman et al. [45] argued that traditional assessment and self-report for people with moderate dementia were not appropriate. Contrary, other researchers state that even people with dementia in advanced stages can participate in interviews and express their preferences [66, 67]. In occupational therapy, critics argue that person-centredness, in terms of client-centredness, should include evaluations from the perspectives of the patients themselves [20]. Including the perspectives of people living with dementia in evaluations of communication is likewise recommended in a recent review [68].

### 6.5. Rehabilitation and People Living with Dementia

Rehabilitation with the guiding principle of person-centredness is recommended for people living with dementia in international health standards [8]. The slim result of this search for evaluations of person-centredness in dementia rehabilitation may be due to the lack of conceptual consensus of rehabilitation for people living with dementia [6]. Researchers describe a reluctance to use the terminology of rehabilitation with regard to multidisciplinary rehabilitative services for people with dementia, because of the progressive nature of the illness and distrust in people with dementias abilities to partake in actions like goalsetting. Instead, terminologies like function-focused care, reablement, restorative care, or goal-oriented care are used [6, 69]. Furthermore, there is not yet consensus as to what rehabilitation for people with dementia entails [6].

Caregivers are often involved in a rehabilitation process and primary caregivers of people with dementia are essential in the daily support [22, 36]. In this study, we focused on the person with dementia in respect of the person with the illness and in line with the current paradigm of person-centredness. In rehabilitation of people with dementia, it may have been more appropriate to include evaluations of proxy respondents.

### 6.6. Strengths and Limitations

This study has illustrated the lack of research-based knowledge evaluating person-centredness in rehabilitation for people living with dementia. However, this study's limitations must be considered when interpreting our result. Relevant articles may have been missed in the review of the literature, as we excluded literature published prior to the year 2000, research published in other languages than English, Norwegian, Swedish, and Danish, and research from nonwestern countries. Furthermore, we excluded grey literature which may hold valuable insights [32], especially in reviews on person-centred approaches [70].

Although we were thorough in our identification of search terms, relevant synonyms for person-centredness could have been missed due to heterogeneity of definitions and understandings of person-centred approaches [18, 56]. Furthermore, evaluation may have been defined too narrowly.

Initially, we viewed the addition of a third search block of rehabilitation or occupational therapy as a strength to systematically answer the research questions [32, 34]. In hindsight, it may have been a major limitation, as interventions related to rehabilitation may be named by other terms such as function-focused care [69], restorative care [71], reablement [72], or habilitation [73].

It may be a limitation that we did not change the research questions or inclusion criteria, when we learnt that only one article could be identified in the review of the literature. We however regarded it interesting and important to make explicit that when reviewing the literature, using these search terms, literature on evaluations of person-centredness in rehabilitation for people living with dementia was lacking.

## 7. Conclusion

Rehabilitation, with person-centredness as a guiding principle, has been recommended to people living with dementia for decades. This study suggests that evaluations of person-centredness in rehabilitation for people living with dementia in peer-reviewed literature are lacking. Only one article could be identified in a review of the literature that evaluated person-centredness in rehabilitation for people living with dementia, and the identified study was in the field of occupational therapy. This gap in research is important because the evaluation and documentation of interactions between people living with dementia and healthcare professionals in rehabilitation are needed to identify effective strategies to pursue and uphold person-centred care.

It was discussed whether a lack of consensus of rehabilitation for people living with dementia could account for the lack of identified literature. Given the dearth of research in the area, this article included research in person-centred dementia care in the discussion, which potentially can inspire practice and research of rehabilitation. To understand the complex nature of person-centredness, a variety of research methodologies of qualitative and quantitative character are recommended for evaluations.

## Figures and Tables

**Table 1:** Methodological quality of the included article.

Author, year, country	Aim	Methods design	Population setting	Result	Quality of study based on CASP
Teitelman et al. [45], 2010, USA	To discuss the potency of the social environment in facilitating occupational engagement in PWD	Qualitative. *Data collection*: interview and observation *Data analysis:* Van Manen's phenomenological analysis	2 *study cases*: 2 PWD (moderate), 2 family members, 2 staff. *Setting*: Therapeutic activity sessions in occupational therapy in a memory support unit	Social therapeutic interactions can promote or inhibit occupational engagement	*Strengths*: Clear aim. Appropriate design. Transparent data collection and data analysis. Ethics considered. Clear statement of findings. Discussion of evidence and credibility of findings. *Weaknesses*: Unclear if some participants chose not to take part. No topic guide for interviews. No details on how research was explained to participants

PWD = people with dementia; CASP = Critical Appraisal Skills Programme.

## Appendix

According to PRISMA guidelines [42], a full electronic search strategy for at least one database, including date of search and limits used, must be presented.

We used the following search strategy, formulated in CINAHL and adapted it to all databases searched: Dementia^*∗*^ OR Alzheimer^*∗*^ OR Lewy Bod^*∗*^ AND Patient Cent^*∗*^ OR Patient-Cent^*∗*^ OR Patient Focus^*∗*^ OR Patient-Focus^*∗*^ OR Person Cent^*∗*^ OR Person-Cent^*∗*^ OR Client Cent^*∗*^ OR Client-Cent^*∗*^ OR Personalised OR Personalised OR Individualised OR Individualised OR Tailored OR Tailor-Made OR Tailor-Made OR Tailormade OR User-Involvement OR User-Involvement OR End User-Involvement OR End-User Involvement OR User-Participation OR User-Participation OR End User-Participation OR End-User Participation OR Patient Participation OR Patient Involvement OR Patient Engagement OR Citizen Involvement OR Citizen Participation OR Consumer Participation OR Consumer Involvement OR Shared Decision-Making∗ OR Shared Decision-Making∗ OR Collaboration OR Partnership∗ OR Goal Setting∗ OR Goal-Setting∗ OR Goalsetting∗ AND Occupational Therap∗ OR Rehabilitation∗ OR Reablement OR Re-ablement OR Restorative Care. Limits: Peer reviewed AND year 2000–14.03.2017 AND Danish OR English OR Norwegian OR Swedish.

### Search History from PubMed

(((((((((((Dementia[Title/Abstract] OR Dementias[Title/Abstract] OR Alzheimer[Title/Abstract] OR Alzheimers[Title/Abstract] OR Alzheimer s[Title/Abstract] OR Lewy body[Title/Abstract] OR Lewy bodies[Title/Abstract]))) OR Dementia, Multi-Infarct[MeSH Terms]) OR Vascular dementia[MeSH Terms]) OR Frontotemporal Dementia[MeSH Terms]) OR Lewy Body Disease[MeSH Terms]) OR Alzheimer disease[MeSH Terms]) OR Dementia[MeSH Terms])) AND (((((Patient-Centered Care[MeSH Terms]) OR Patient Participation[MeSH Terms]) OR Person-Centered Therapy[MeSH Terms]) OR Decision Making[MeSH Terms]) OR ((Patient Centered[Title/Abstract] OR Patient Centred[Title/Abstract] OR Patient Centeredness[Title/Abstract] OR Patient Centredness[Title/Abstract] OR Patient Focus[Title/Abstract] OR Patient Focused[Title/Abstract] OR Person Centered[Title/Abstract] OR Person Centred[Title/Abstract] OR Person Centeredness[Title/Abstract] OR Person Centredness[Title/Abstract] OR Client Centered[Title/Abstract] OR Client Centred[Title/Abstract] OR Client Centeredness[Title/Abstract] OR Client Centredness[Title/Abstract] OR Personalised[Title/Abstract] OR Personalized[Title/Abstract] OR Individualised[Title/Abstract] OR Individualized[Title/Abstract] OR Tailored[Title/Abstract] OR Tailor Made[Title/Abstract] OR Tailormade[Title/Abstract] OR User Involvement[Title/Abstract] OR End User Involvement[Title/Abstract] OR User Participation[Title/Abstract] OR End User Participation[Title/Abstract] OR Patient Participation[Title/Abstract] OR Patient Involvement[Title/Abstract] OR Patient Engagement[Title/Abstract] OR Citizen Involvement[Title/Abstract] OR Citizen Participation[Title/Abstract] OR Consumer Participation[Title/Abstract] OR Consumer Involvement[Title/Abstract] OR Shared Decision Making[Title/Abstract] OR Shared Decision Makings[Title/Abstract] OR Collaboration[Title/Abstract] OR Partnership[Title/Abstract] OR Partnerships[Title/Abstract] OR Goal Setting[Title/Abstract] OR Goal Settings[Title/Abstract] OR Goalsetting[Title/Abstract] OR Goalsettings[Title/Abstract])))) AND ((((((Occupational Therapy[Title/Abstract] OR Occupational Therapies[Title/Abstract] OR Rehabilitation[Title/Abstract] OR Rehabilitations[Title/Abstract] OR Reablement[Title/Abstract] OR Re-ablement[Title/Abstract] OR Restorative Care[Title/Abstract]))) OR rehabilitation center[MeSH Terms]) OR Rehabilitation[MeSH Terms]) OR Occupational Therapy[MeSH Terms]) AND ((“2000/01/01”[PDat] : “3000/12/31”[PDat]) AND (Danish[lang] OR English[lang] OR Norwegian[lang] OR Swedish[lang])).
